# Analysis of clock gene-miRNA correlation networks reveals candidate drivers in colorectal cancer

**DOI:** 10.18632/oncotarget.9989

**Published:** 2016-06-14

**Authors:** Gianluigi Mazzoccoli, Tommaso Colangelo, Anna Panza, Rosa Rubino, Cristiana Tiberio, Orazio Palumbo, Massimo Carella, Domenico Trombetta, Annamaria Gentile, Francesca Tavano, Maria Rosa Valvano, Clelia Tiziana Storlazzi, Gemma Macchia, Angelo De Cata, Giovanni Bisceglia, Daniele Capocefalo, Vittorio Colantuoni, Lina Sabatino, Ada Piepoli, Tommaso Mazza

**Affiliations:** ^1^ Division of Internal Medicine and Chronobiology Unit, IRCCS “Casa Sollievo della Sofferenza”, San Giovanni Rotondo (FG), Italy; ^2^ Department of Sciences and Technologies, University of Sannio, Benevento, Italy; ^3^ Division of Gastroenterology and Research Laboratory, IRCCS “Casa Sollievo della Sofferenza”, San Giovanni Rotondo (FG), Italy; ^4^ Medical Genetics Service, IRCCS “Casa Sollievo della Sofferenza”, San Giovanni Rotondo (FG), Italy; ^5^ Oncology-Research Laboratory, IRCCS “Casa Sollievo della Sofferenza”, San Giovanni Rotondo (FG), Italy; ^6^ Department of Biology, University of Bari, Bari, Italy; ^7^ Department of Surgical Sciences, IRCCS “Casa Sollievo della Sofferenza”, San Giovanni Rotondo (FG), Italy; ^8^ Bioinformatics Unit, IRCCS “Casa Sollievo della Sofferenza”, San Giovanni Rotondo (FG), Italy; ^9^ Division of Epidemiology and Health Statistics, IRCCS “Casa Sollievo della Sofferenza”, San Giovanni Rotondo (FG), Italy

**Keywords:** miRNA-mRNA, clock genes, circadian, colorectal cancer

## Abstract

Altered functioning of the biological clock is involved in cancer onset and progression. MicroRNAs (miRNAs) interact with the clock genes modulating the function of genetically encoded molecular clockworks. Collaborative interactions may take place within the coding-noncoding RNA regulatory networks. We aimed to evaluate the cross-talk among miRNAs and clock genes in colorectal cancer (CRC). We performed an integrative analysis of miRNA-miRNA and miRNA-mRNA interactions on high-throughput molecular profiling of matched human CRC tissue and non-tumor mucosa, pinpointing core clock genes and their targeting miRNAs. Data obtained *in silico* were validated in CRC patients and human colon cancer cell lines. *In silico* we found severe alterations of clock gene–related coding-noncoding RNA regulatory networks in tumor tissues, which were later corroborated by the analysis of human CRC specimens and experiments performed *in vitro*. In conclusion, specific miRNAs target and regulate the transcription/translation of clock genes and clock gene-related miRNA-miRNA as well as mRNA-miRNA interactions are altered in colorectal cancer. Exploration of the interplay between specific miRNAs and genes, which are critically involved in the functioning of the biological clock, provides a better understanding of the importance of the miRNA-clock genes axis and its derangement in colorectal cancer.

## INTRODUCTION

Colorectal cancer (CRC) represents the third most commonly diagnosed malignancy and the fourth leading cause of cancer deaths [1–3]. At present, the only therapeutic strategies include surgery and adjuvant or neo-adjuvant chemotherapy; thus, there is a definite need for innovative biological targets for prognostic stratification and therapeutic interventions. Colorectal carcinogenesis implies alterations of basic cellular processes, such as differentiation, proliferation and apoptosis. These processes and the overall physiologic functions of living beings are characterized by rhythmic oscillations recurring with a periodicity of approximately 24 hours (circadian) [4–7]. The circadian rhythms are driven by molecular clockworks ticking in every cell through transcriptional-translational feedback loops (TTFLs) revolving in about 24 hours and operated by circadian genes and proteins [8, 9]. TTFLs are initiated by the transcription factors BMAL1/2 (ARNTL/ARNTL2) and CLOCK/NPAS2, which heterodimerize and activate the transcription of PERIOD (*PER1-3*) and CRYPTOCHROME (*CRY1-2*) genes. In turn, PER and CRY proteins heterodimerize and inhibit the transcriptional activity of BMAL1/2 - CLOCK/NPAS2 heterodimers [10]. BMAL1/2 activates also the expression of *NR1D1/NR1D2*, encoding the transcription factors Rev-Erbα/β that, in turn, negatively control the rhythmic transcription of *BMAL1*, hindering RORα binding to the response element in the *BMAL1* promoter region [11]. In the molecular clockwork of *Drosophila Melanogaster* a key role is played by *Timeless* (*Tim*) [12], and precisely there are two *Tim* paralogs, *Tim1* and *Tim2* (also called *Timeout*). *Tim1* is a canonical circadian component, whereas the role of *Tim2* in the fly is presently unclear [13]. In mammals the homolog of *Tim2* seems to be not essential for the ticking of the biological clock, but the encoded protein interacts with TIPIN and is crucial for replication protection and genomic stability [14], as well as for coordination of mitotic kinase activation with DNA replication termination [15], period determination and DNA damage-dependent phase advancing of the circadian clock [16]. The circadian proteins undergo also post-translational modifications, such as phosphorylation by Casein Kinase 1ε (CK1ε) and AMPK and deacetylation by SIRT1, which tag them for degradation or modify their functions, respectively [17–20].

MicroRNAs (miRNAs) are small, endogenous, single-stranded RNAs of 18–22 nucleotides (nt) in length that are emerging as important modulators of gene expression at post-transcriptional level. They promote translational repression and/or destabilization of target messenger RNAs (mRNAs) by base-pairing to partially complementary sites in the 3′ untranslated region (3′UTR), less frequently in the coding sequences (CDS), of the target mRNAs [21]. The same mRNA transcript may be targeted by different miRNAs and a single miRNA, in turn, may target several different mRNAs [22]. Derangements in the miRNA-mRNA interactions [23], as well as altered expression of clock genes [24, 25] play a role in CRC onset and development and impact colon cancer cell behavior and response to chemotherapy [26, 27].

In this study we performed an integrative analysis of miRNA-miRNA and miRNA-mRNA interactions using high-throughput mRNA and miRNA expression profiling of CRC datasets to explore miRNA-clock genes interplays searching for functional relationships that are deranged in this tumor type. We addressed this aim by investigating the miRNA correlation network built on a miRNA expression profile database, which was populated with a set of human CRC specimens and matched adjacent normal mucosa in order to identify the miRNAs that, among those targeting the core clock genes, may play a key role in this malignancy. These miRNAs populated the subset 1 of this analysis. Similarly, we investigated the miRNA correlation network built on the miRNA expression profiles obtained from the Colon adenocarcinoma (COAD) database publicly available on the web through The Cancer Genome Atlas (TCGA) Data Portal (https://tcga-data.nci.nih.gov/tcga/, downloaded March 15^th^, 2015). These miRNAs populated the subset 2 of this analysis. We then compared the two correlation networks in terms of connectivity and node centrality. Finally, based on the expression levels and on topological considerations of miRNAs and clock genes, we selected miR-139-5p and *TIMELESS* to be further validated, *in vitro*, in a new cohort of CRC patients and in human colon cancer cell lines.

## RESULTS

### A priori filtering of miRNA correlation pairs in TCGA and colon cancer microarray cohorts

We performed the Pearson Product Moment Correlation (PPMC) analysis on our CRC dataset (Table 1) and one publicly available dataset: miRNAs making significant pairs were counted and the numbers obtained were compared. The miRNAs detected in the COAD-TCGA dataset and present or not in our arrays were reported. Notably, COAD-TCGA control and tumor samples shared 501 miRNAs, while all miRNAs in control samples were shared with tumor samples. Moreover, among these latter samples, half of individual miRNAs were common to both datasets. The numbers of miRNAs that were exclusively correlated in control rather than in tumor samples were almost balanced in the microarray dataset (Supplementary Tables S1–S5).

**Table 1:** Clinical characteristics of the colorectal cancer patients examined for the microarray analysis

No.		Age (years)	Sex	Cancer site	Adenocarcinoma	Histological grade	Clinical stage	TNM classification
1	PS1	58	Female	PTRCF	Yes	G2	II	T3N0M0
2	PS3	81	Male	PTRCF	Yes	G1	I	T2N0M0
3	PS4	61	Male	Sigmoid	Yes	G2	III	T3N1M0
4	PS5	69	Female	DTLCF	Yes	G1	IV	T4N2M1
5	PS7	77	Male	Sigmoid	Yes	G2	I	T2N0M0
6	PS10	67	Male	Sigmoid	Yes	G2	III	T3N2M0
7	PS11	67	Male	Sigmoid	Yes	G1	III	T3N1M0
8	PS12	57	Male	Sigmoid	Yes	G2	II	T3N0M0
9	PS13	81	Female	Sigmoid	Yes	G1	II	T3N0M0
10	PS14	81	Female	Rectosigmoid	Yes	G2	I	T2N0M0
11	PS15	77	Male	DTLCF	Yes	G2	III	T3N1M0
12	PS17	73	Female	Coecum	Yes	G3	III	T3N1M0
13	PS19	57	Male	Sigmoid	Yes	G2	II	T3N0M0
14	PS20	55	Male	Descendens	Yes	G1	II	T3N0M0

Abbreviations: PTRCF = Proximal transverse and right colic flexure; DTLCF = Distal transverse and left colic flexure TNM (classification of malignant tumors): T, size or direct extent of the primary tumor; N, degree of spread to regional lymph nodes; M, presence of distant metastasis.

### Identification of common or unique miRNA pairs through different conditions

**Figure 1:**
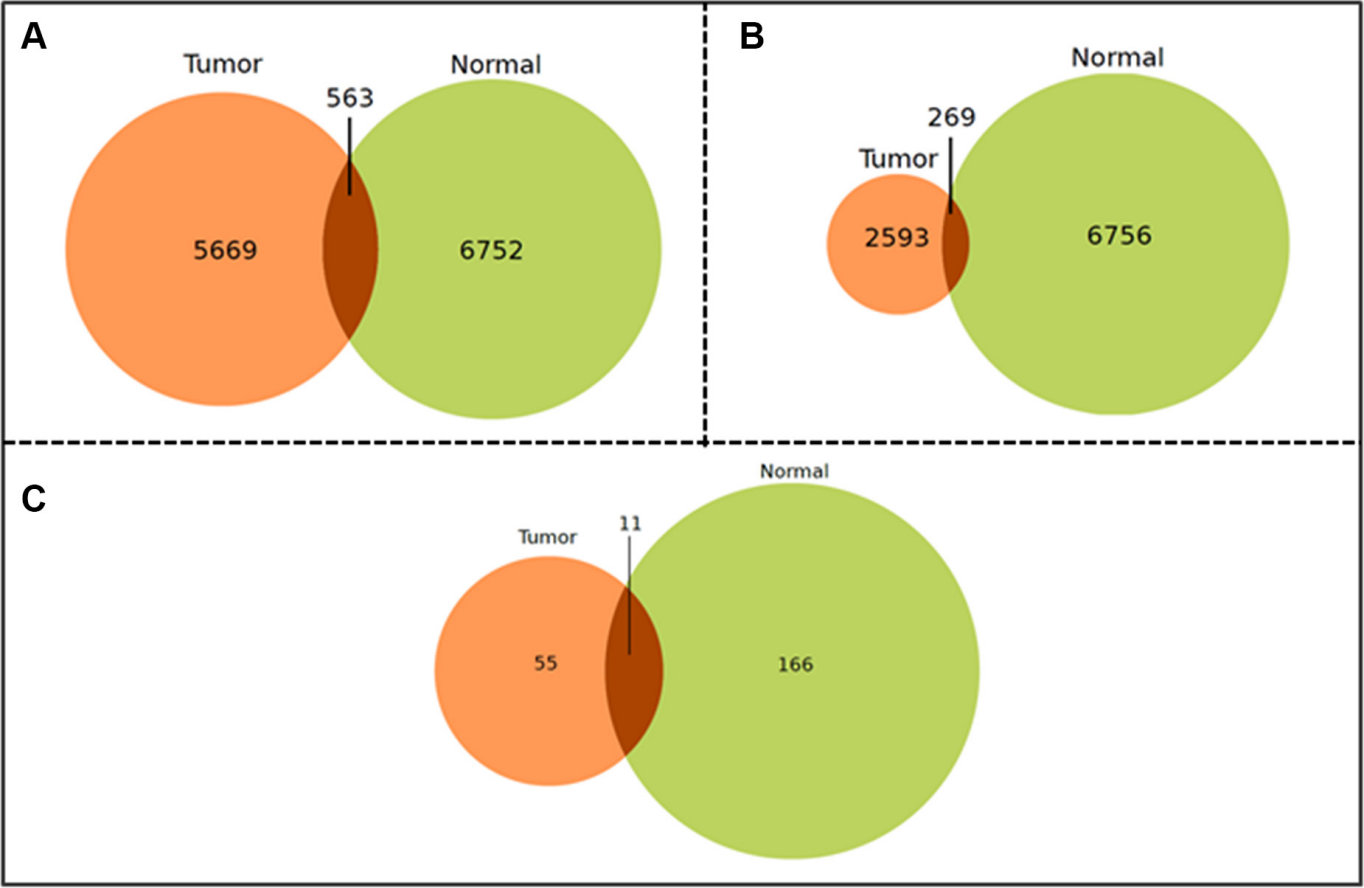
Venn-Diagrams of the shared miRNA-miRNA correlation pairs Venn-Diagram of the shared miRNA-miRNA correlation pairs in the microarray (**A**) and the COAD-TCGA (**B**) datasets that are exclusive to tumor tissue (left), exclusive to non-tumor tissue (right) or common between the two tissue types (overlapping). (Filtering criteria: Pearson Product Moment Correlation score ± 0.6, absolute value, and *p* ≤ 0.05); (**C**) Venn-Diagram of the shared miRNA-miRNA correlation pairs in the microarray dataset. (Filtering criteria: Pearson Product Moment Correlation score ± 0.8, absolute value, and *p* ≤ 0.05).

Weak correlations were excluded by considering only miRNA pairs with an r exceeding ± 0.8. Thus, the remaining pairs of miRNAs in tumor and control networks were compared to discover those peculiar for each disease condition. Figure 1A shows the intersections of these miRNA correlation pairs in the microarray dataset. It is worthy of note that the COAD-TCGA dataset (Figure 1B) is unbalanced in terms of number of tumor correlation pairs with respect to the number of control correlation pairs. Roughly, 10% of pairs are in common with the control ones in both datasets (Figure 1C). MiRNAs targeting clock genes were searched in both subsets with the main goal to define whether these miRNA groups had some elements in common. Indeed, a significant percentage of them was present in each subset and a further interesting part correlated (i.e., there were correlation pairs between miRNAs targeting clock genes). This finding likely indicates that pairs of correlated miRNAs may cooperate to target the core clock genes. Moreover, the strongly correlated miRNAs set was bigger in control than tumor samples, underscoring, on one hand, the important regulatory role of miRNAs in normal tissue and, on the other hand, that deregulation of almost one third of them may play a critical role in CRC development.

### Candidate miRNAs involved in clock genes deregulation in colorectal cancer

**Figure 2:**
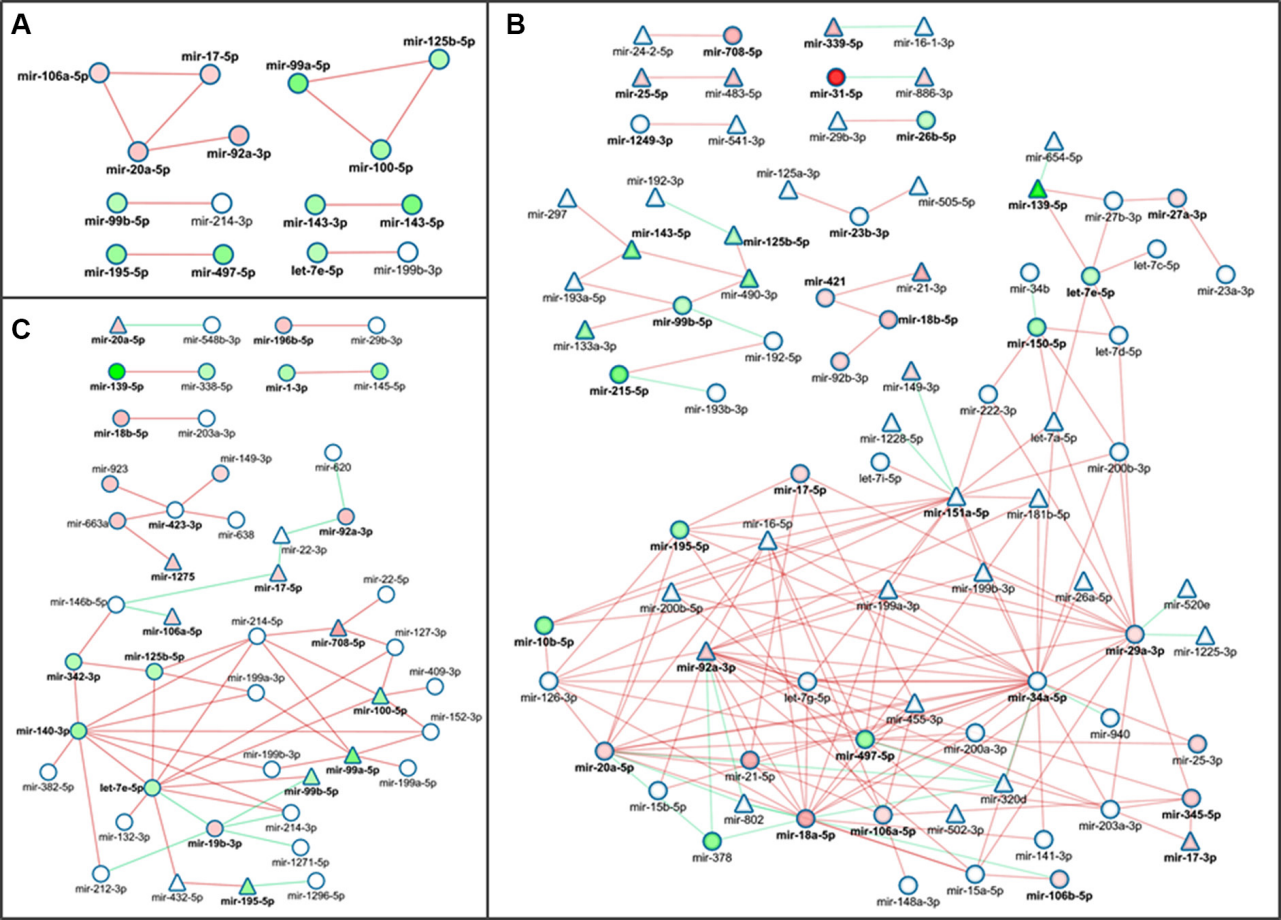
Network picture of colorectal cancer miRNA-ome datasets based on Pearson Product Moment Correlation network of commonly correlated miRNAs in tumor and non-tumor tissue (**A**) Network representation of the common miRNA correlation pairs among neoplastic (tumor) and matched non-tumor tissue (control). (**B**) Network visual representation of the miRNA correlation pairs specific for the control tissue. (**C**) Network visual representation of the miRNA correlation pairs specific for the tumor tissue. Nodes represent miRNAs that are linked by an edge if their expression values correlate (red for positive correlations, green otherwise). Shapes and colours represent features of nodes and edges. Red nodes render up-regulated miRNAs in tumor; green nodes render down-regulated miRNAs in tumor. Bold node render miRNAs that are experimentally known to target any core clock gene. Triangle-shaped nodes render miRNAs whose expression values are cross-checked in the TCGA-COAD dataset.

We drew and analyzed the networks of the following subsets: the common subset, the correlation subset exclusive for the correlation pairs in non-tumor tissue (control subset) and the correlation subset exclusive for the correlation pairs in neoplastic tissue (tumor subset). The visual representation of these networks is rendered in Figure 2. From a preliminary survey, it can be noticed that the common subset miRNAs generate a highly sparse correlation network and tend to form closed and unrelated clusters (Figure 2A). miR-20a-5p, the miRNA with the highest betweeness centrality value (0.667), reaches the maximum degree in this network that is 3. It is important to consider that, in this network, many miRNAs are also differentially expressed without a significant link between the fold-change and the *r* value. Moreover, many miRNAs targeting clock genes did not come out from the COAD-TCGA dataset analysis.

Analysis of the miRNA network built on the control subset verified a strong correlation among the vast majority of the nodes, with only a few tending to form small isolated clusters (Figure 2B). Several positive correlations were present in the core network, many of which did not appear in the COAD-TCGA dataset. The node with the smallest average shortest path length is miR-34a-5p that targets CRY1, a core element of the molecular clockwork. This miRNA came out from the analysis of the COAD-TCGA dataset and displayed the highest degree centrality and the lowest average shortest path length together with a relatively low clustering coefficient. However, the expression of this miRNA did not significantly change between tumor and non-tumor tissues. On its side, the miRNA network built on the tumor subset displayed some interesting properties (Figure 2C). Apart from isolated correlation pairs, it showed an intricate correlation network and the node with the highest degree was let-7e-5p that was only slightly down-regulated in tumor tissue. This miRNA, belonging to the let family and involved in stemness, showed also a good centrality value (around 0.4), pointing it as a hub in our tumor correlation network. Moreover, this node was positively correlated with a series of other miRNAs hallmarked by low-fold change values, implying that their deregulation might trigger the reduced expression of several other miRNAs. Additionally, the fold-change and the correlation values indicated miR-19-3b as a possible negative regulator of the let-7-e-5p gene, as it was negatively correlated and up-regulated in tumor. miR-19b-3p targets *TIMELESS* and *NR1D2* and many of its correlated neighbours, let-7e-5p, miR-99b-5p and, indirectly, miR-140-3p (separated by 1 degree and connected via miR-214-3p and miR-212-3p) are instead down-regulated, suggesting a potential regulation of miR-19b-3p on these miRNAs.

**Figure 3:**
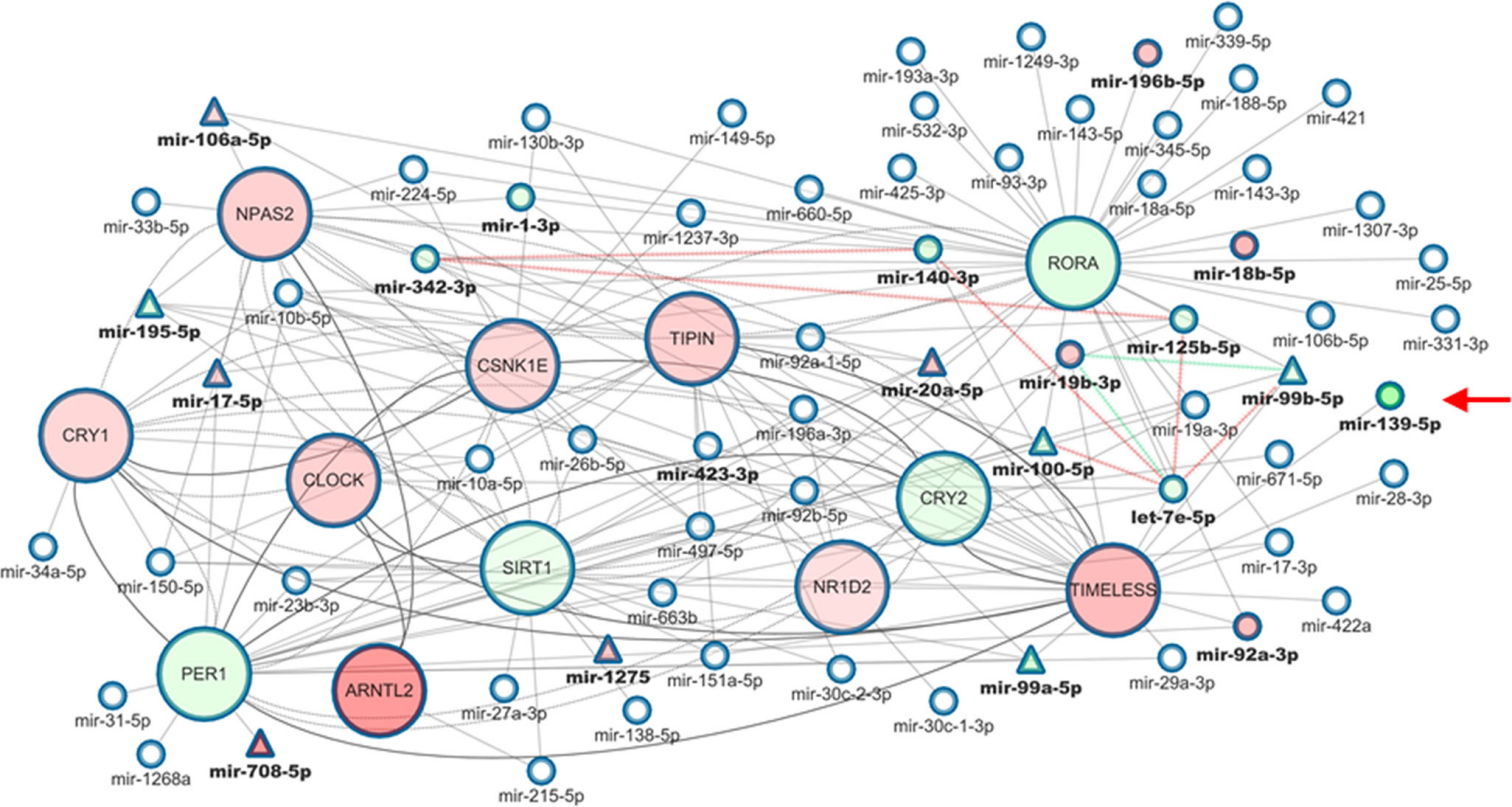
Core clock genes-miRNAs correlation network in colorectal cancer Multi-layer network of clock genes and targeting miRNAs in which nodes represent clock genes or miRNAs, while edges represent correlation or interaction among miRNAs or genes. Nodes representing genes are bigger than miRNA nodes. miRNA names are bolded if any association with clock genes is known. miRNA nodes are represented as triangles if their expression values are consistent in the COAD-TCGA dataset. Red nodes are up-regulated genes/miRNAs in tumor tissue, green nodes are down-regulated genes/miRNAs in tumor tissue. Edges between genes are solid or dashed, depending on whether they represent direct or indirect interactions, as reported in BioGRID. Edges connecting miRNAs-genes render targeting interactions as from miRWalk 2.0. Arrowed edges connecting two miRNAs represent strong positive (> 0.8) or negative (≤ 0.8) correlations between them, and are colored in red or green, respectively.

As illustrated in Table 2 and Figure 3, miR-125b-5p, miR-140-3p, let-7e-5p and miR-99b-5p are all down-regulated in tumor as compared to non-tumor tissues and all target clock genes. Noteworthy, miR-19b-3p is weakly (−0.47) anti-correlated with miR-99b-5p in the control subset, but the anti-correlation strength becomes stronger in the tumor subset. Besides, these miRNAs are involved in the pathways controlling cancer cell proliferation and invasiveness (Supplementary Table S6)

**Table 2:** List of the clock genes targets of the most relevant correlated miRNAs

	Fold Change (Tumor versus Control)	Targets
let-7e-5p	Down	PER1, CLOCK
miR-125b-5p	Down	PER1, RORA, TIPIN
miR-140-3p	Down	NPAS2, TIMELESS
miR-99b-5p	Down	PER1, RORA, TIMELESS
miR-19b-3p	Up	TIMELESS, NR1D2
miR-139-5p	Down	TIMELESS

In the control network, let-7e-5p is linked to miR-139-5p, a candidate tumor suppressor targeting *TIMELESS* and found down-regulated in many cancer types, but in the tumor network this important link was lost. Interestingly, as reported in Supplementary Table S3, miR-139-5p was down-regulated and ranked among the most deregulated miRNAs in tumor tissues (False Discovery Rate = 0.002668, Fold Change = −6.801375, *p* value = 0.000013). miR-139-5p recognizes two seed regions in *TIMELESS* mRNA and we decided to select this miRNA/gene pair for validation in an independent cohort of patients and, then, for *in vitro* functional studies.

## TIMELESS MRNA AND MIR139-5P LEVELS ARE ALTERED AND INVERSELY CORRELATED IN SPORADIC CRCS

**Figure 4:**
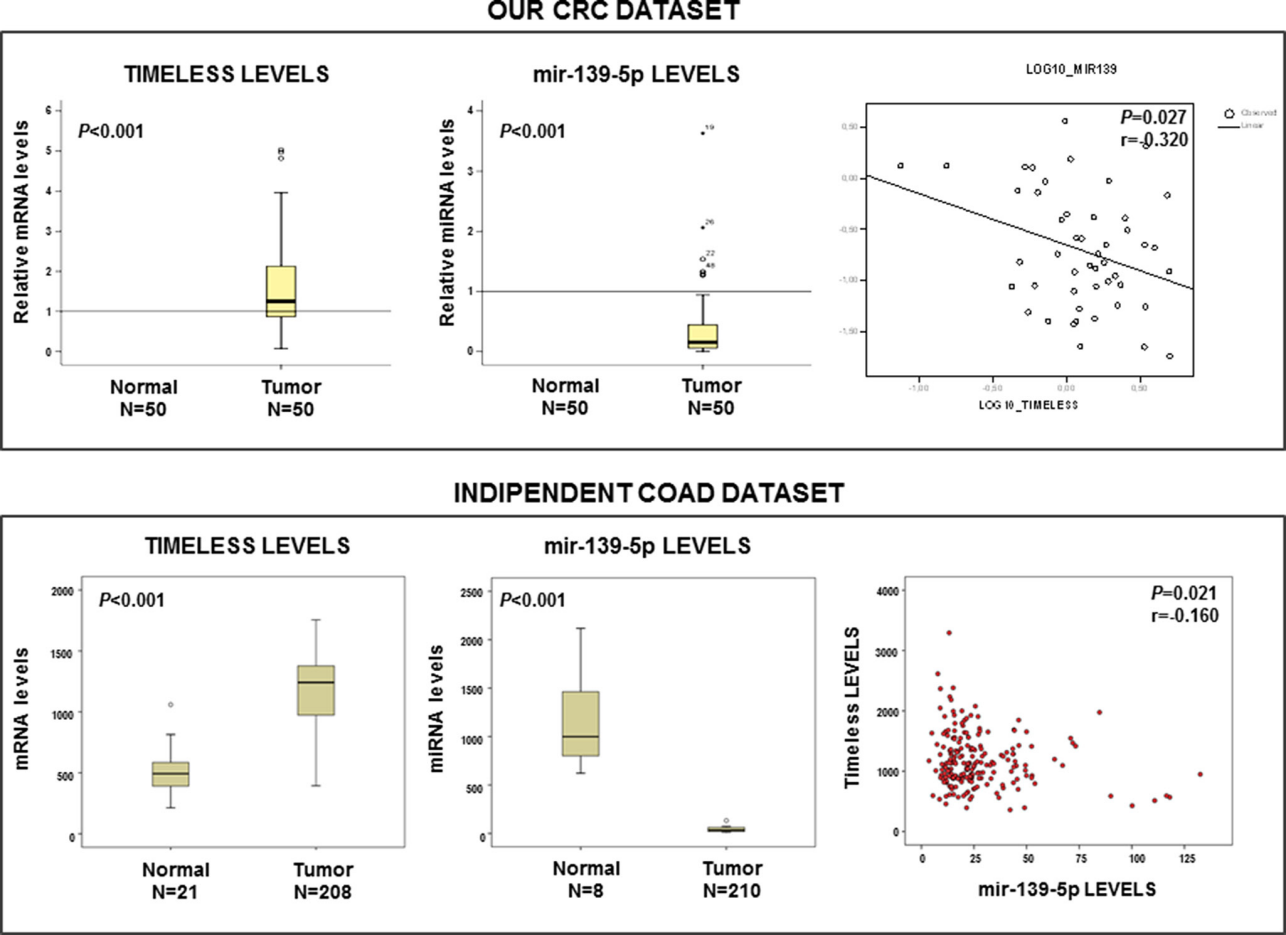
*TIMELESS* mRNA and miR-139-5p levels are inversely correlated in sporadic colorectal cancer Box-plot analysis of *TIMELESS* mRNA and miR-139-5p levels in our colorectal cancer (CRC) patients validation cohort (upper panel) and in the COAD-TCGA dataset (lower panel); *p* value and *r* coefficient of correlation are reported in the panels.

We quantified *TIMELESS* mRNA and miR-139-5p levels in 50 matched pairs of tumor tissues/non-tumor mucosa sampled from 46 well-moderately differentiated (G1-G2) and from 4 poorly differentiated-undifferentiated (G3-G4) CRCs. Median values, 25th (or first quartile, Q1) and 75th percentile (or third quartile, Q3) of *GAPDH*-Ct value/target gene-*C*_t_ value are shown in Figure 4. Compared to matched non-tumor tissues a statistically significant increase of *TIMELESS* mRNA (median = 1.25, Q1-Q3 = 0.87-2.13, *p* < 0.001) and a decrease of miR-139-5p levels (median = 0.15, Q1–Q3 = 0.06–0.44, *p* < 0.0001) was observed in tumor tissues. *TIMELESS* mRNA was inversely correlated with miR-139-5p expression by a Pearson's correlation test (*r* = −0.320; *p* = 0.027) (Figure 4 upper panel). These results were corroborated by the analysis of an independent publicly available COAD-TGCA cohort comprising 210 CRC samples for which mRNA and miRNA expression profile were attainable (https://tcga-data.nci.nih.gov/tcga) (*r* = −0.160; *p* = 0.021) (Figure 4 lower panel).

### Clinical-pathological features of CRC patients and association with TIMELESS mRNA and miR-139-5p levels

Genotype-phenotype associations were evaluated in our validation cohort of CRC patients. After splitting subjects at the median value of gene expression levels, the increased *TIMELESS* mRNA level in tumor tissue was strongly associated with disease spreading, in particular with proximal and distal lymph nodes involvement (*p* = 0.010) and III-IV TNM stages (*p* = 0.017). Lower miR-139-5p levels were associated with a non-mucinous adenocarcinoma histologic type (*p* = 0.035) and high microsatellite instability frequency (*p* = 0.035) (Table 3).

**Table 3:** Association of TIMELESS mRNA and miR-139.5p levels with clinical and pathological features of colorectal cancer patients included in the validation cohort

			TIMELESS				miR139.5p			
		*N* = 50	Median	25th percentile	75th percentile	*p* value	Median	25th percentile	75th percentile	*p* value
Age (years) Mean ± SD 67.2 ± 11.6	< 62	16	1.199	1.061	3.695	0.457	0.122	0.052	0.287	0.360
	62–74	19	1.438	0.606	1.939		0.152	0.091	1.268	
	> 74	15	1.219	0.866	1.796		0.181	0.042	0.408	
Gender	Male	34	1.353	0.974	2.218	0.546	0.224	0.088	0.727	0.050
	Female	16	1.126	0.730	1.927		0.110	0.039	0.195	
Tumor location	Proximal colon †	17	1.163	0.713	1.916	0.419	0.223	0.098	0.755	0.585
	Transverse colon	6	1.247	0.545	1.584		0.157	0.049	0.391	
	Distal colon ‡	27	1.267	0.974	2.324 9		0.121	0.057	0.444	
Grading	G1/G2	45	1.267	0.866	2.218	0.683	0.181	0.056	0.679	0.706
	G3/G4	5	1.163	1.119	1.572		0.132	0.098	0.152	
Modified Dukes Staging System	A	4	1.308	0.977	1.585	0.167	0.047	0.003	0.239	0.265
	B	20	0.989	0.564	1.699		0.202	0.068	1.096	
	C	24	1.714	1.127	2.981		0.166	0.085	0.495	
	D	2	1.396	1.219	1.572		0.092	0.053	0.132	
Histologic Type adeno carcinoma	Mucinous	5	1.854	1.063	1.939	0.615	0.727	0.224	0.943	0.035
	Non mucinous	45	1.235	0.866	2.129		0.132	0.056	0.408	
Microsatellite instability frequency	missing	8				0.381				0.035
	High	9	1.543	1.159	1.916		0.053	0.040	0.098	
	Low	10	1.556	1.163	2.129		0.219	0.111	0.416	
	Stable	19	1.235	0.974	1.939		0.209	0.056	0.943	
	LOH	4	0.789	0.432	2.127		0.574	0.203	1.125	
Depth of tumor invasion	T2	4	1.308	0.977	1.585	0.543	0.047	0.003	0.239	0.225
	T3	43	1.267	0.747	2.324		0.181	0.057	0.679	
	T4	3	1.119	0.074	1.572		0.132	0.079	1.326	
Lymph node involvement	N0	24	0.989	0.594	1.564	0.010	0.132	0.041	0.684	0.543
	N1	14	1.535	1.119	1.939		0.166	0.098	0.755	
	N2	12	2.613	1.243	3.695		0.170	0.054	0.240	
TNM stage	I-II	27	1.004	0.582	1.584	0.017	0.151	0.079	0.312	0.899
	III-IV	23	1.632	1.135	2.590		0.152	0.042	0.924	
Vascular invasion	Not	27	1.159	0.636	1.939	0.365	0.181	0.057	0.679	0.992
	Yes	23	1.267	1.063	3.371		0.140	0.056	0.391	

† caecum and ascending colon;

‡ descending colon, sigmoid colon and rectum; LOH = loss of heterozigosity; N0 = no lymph node involvement N1 = proximal lymph node involvement; N2 = distal lymph node involvement; TNM (classification of malignant tumors): T, size or direct extent of the primary tumor; N, degree of spread to regional lymph nodes; M, presence of distant metastasis.

No statistically significant difference was found for survival rates in a Kaplan–Meier analysis of censored data after stratifying patients according to the median expression value of *TIMELESS* mRNA (Log Rank–Mantel-Cox test *p* = 0.721) and miR139-5p (Log Rank-Mantel-Cox test *p* = 0.515) (Supplementary Figure S1).

### TIMELESS and miR-139-5p levels are inversely related in human colon cancer cell lines

**Figure 5:**
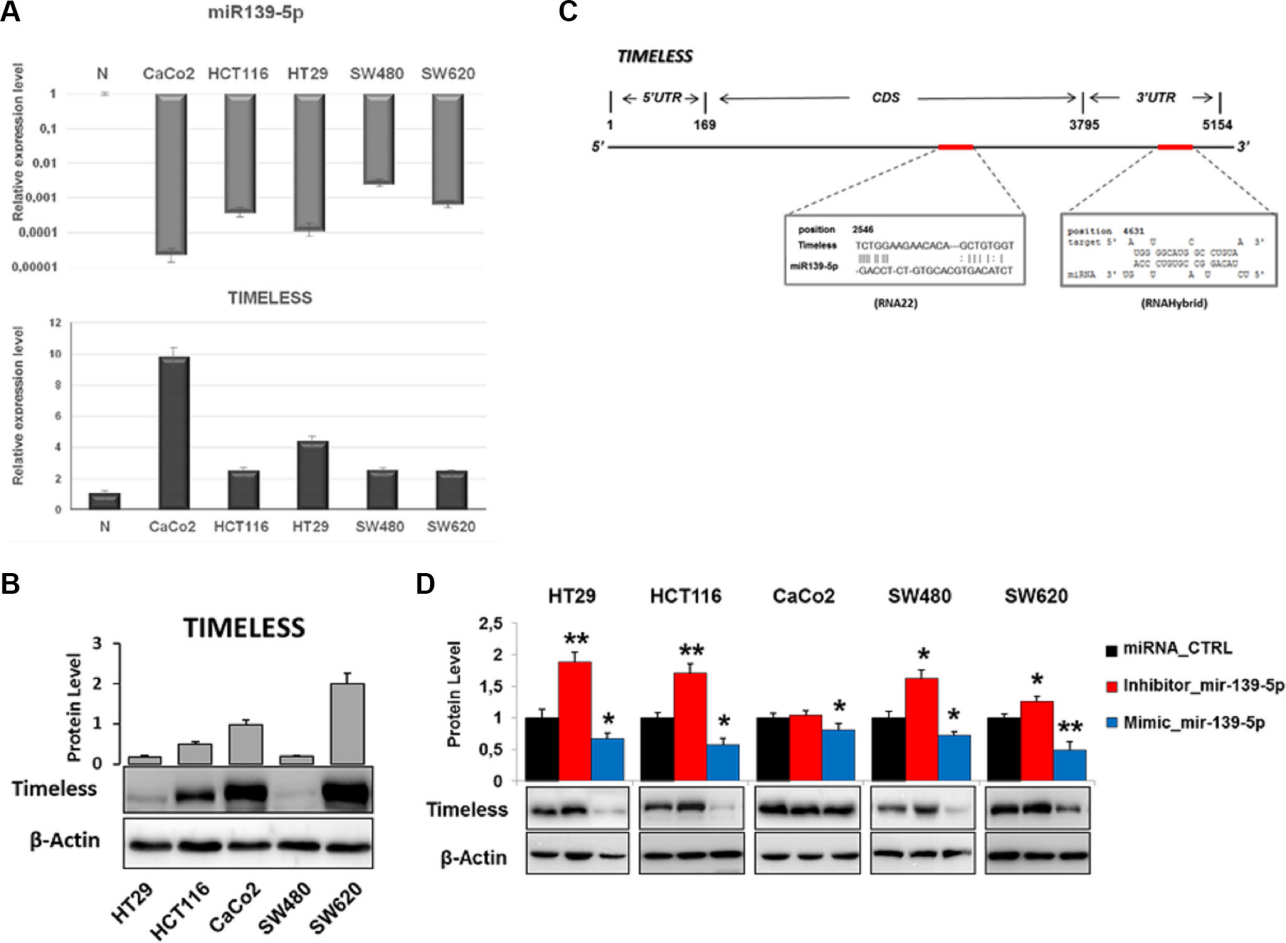
miR-139-5p negatively regulates *TIMELESS* expression in human colon cancer cell lines (**A**) The bar plot reports miR-139-5p and *TIMELESS* mRNA levels evaluated by qRT-PCR analysis in the indicated cell lines with respect to the average expression detected in non-tumor tissues. (**B**) Immunoblot detection of TIMELESS protein in five human colon cancer cell lines; relative fold-change, obtained by densitometric analysis and normalization to β-actin, is reported in the corresponding histograms. (**C**) Schematic representation of human *TIMELESS* mRNA: two specific highly conserved miR-139-5p binding sites are located in the coding region (CDS; RNA22 prediction) and in 3′ UTR (RNAhybrid prediction). (**D**) Western blot analysis of TIMELESS in cells transfected with a miR-139-5p mimic or inhibitor, or scrambled controls; the histogram reports the protein fold-change with respect to scrambled-transfected cells. Samples were analyzed in triplicate and data are reported as mean ± S.D. and representative of three independent experiments. *p ≤ 0.05; **p ≤ 0.01.

*TIMELESS* expression at the mRNA and protein levels was evaluated in CaCo2, HCT116, HT29, SW480 and SW620 cells: *TIMELESS* mRNA expression was higher in tumor than non-tumor mucosa and mainly in CaCo2 and HT29 cells with respect to HCT116, SW480 and SW620 cells (Figure 5A). Western blot analysis on extracts from the same cells showed higher levels of the protein in CaCo2, HCT116 and in particular SW620 cells, suggesting a key role played by miR-139-5p in *TIMELESS* post-transcriptional regulation (Figure 5B). Indeed, miR-139-5p expression displayed an opposite pattern to TIMELESS, with lower levels in all the examined cell lines than non-tumor mucosa; lower levels were detected in CaCo2 and HT29 with respect to HCT116, SW480 and SW620 cells (Figure 5A). Bioinformatic analysis identified two seed regions for miR-139-5p in the CDS and 3′UTR of *TIMELESS* mRNA, respectively (Figure 5C). Thus, we investigated the miR-139-5p-dependent TIMELESS regulation *in vitro*. Modulation of miR-139-5p expression through mimic or antisense RNAs induced remarkable and inverse variations of TIMELESS in CRC cells. In detail, miR-139-5p inhibition resulted in TIMELESS overexpression in HCT116 and HT29 with respect to SW480 and SW620, while no statistically significant variation was observed in CaCo2 cells. In contrast, transfection of a miR-139-5p mimic down-regulated TIMELESS in all cells lines under investigation (Figure 5D). These data confirm that miR-139-5p inversely correlates with TIMELESS regulating its protein levels in human colon cancer cells.

### FISH comparative analysis of TIMELESS copy numbers in human colon cancer cell lines

**Figure 6:**
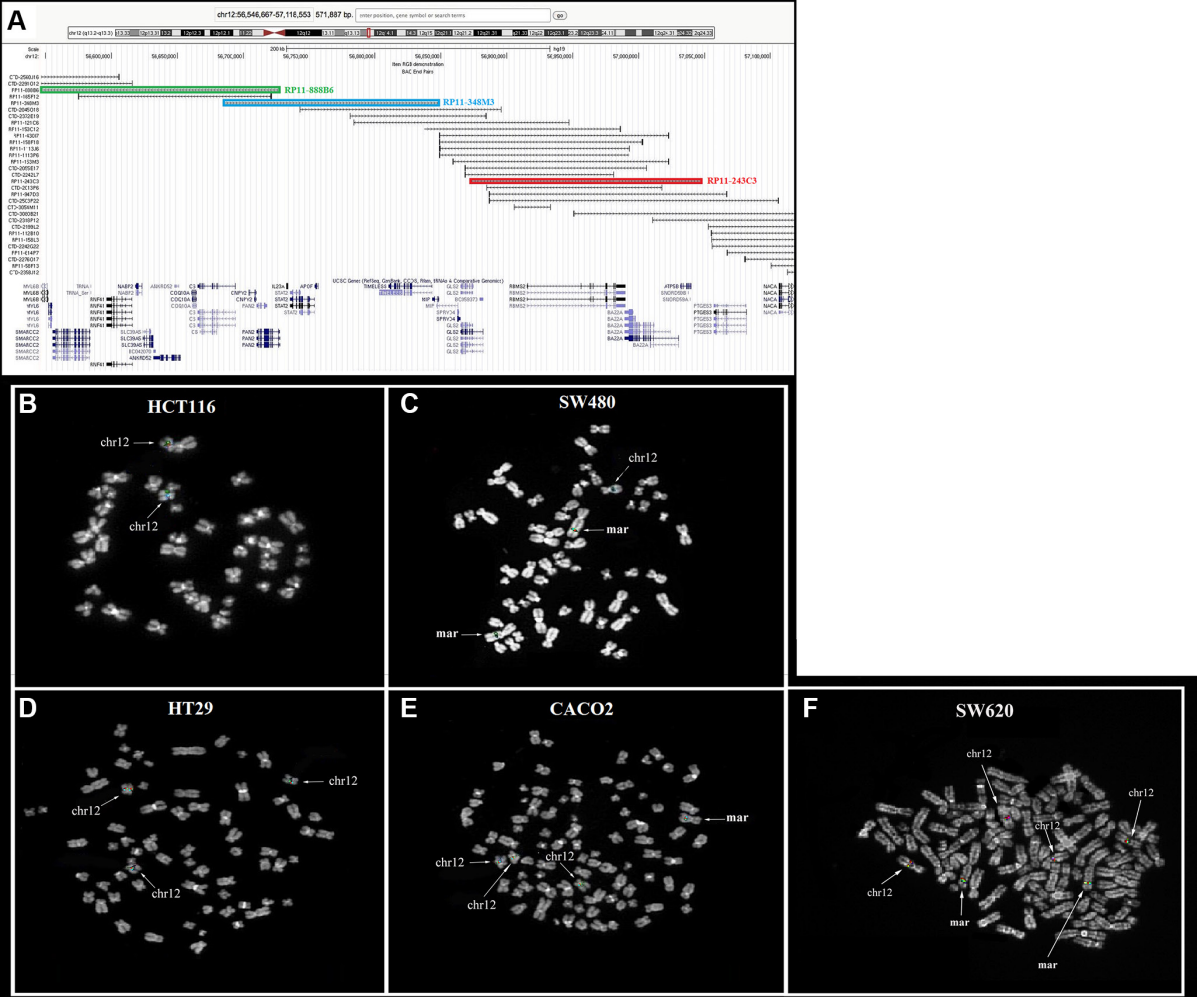
Comparative analysis of *TIMELESS* copy numbers performed by FISH in human colon cancer cell lines (**A**) The BAC probe set used for the screening of TIMELESS copy number gains. The colored rectangles (blue, red, and green) correspond to the FISH results obtained respectively for probes RP11-348M3, RP11-243C3, and RP11-888B6. Genes are reported in blue at the bottom of the figure. (B–F) FISH analysis results. In HCT116 the three probes gave signals on the two normal chromosome 12 homologs (**B**); in SW480, two markers showed positivity for all the probes used, as well as one normal chromosome 12 (**C**); HT29 displayed a whole chromosome 12 trisomy (**D**); CACO2 showed the positivity of a marker chromosome of unknown origin, in addition to chromosome 12 trisomy (**E**); SW620 displayed a whole-chromosome 12 tetrasomy and the positivity of two additional marker chromosomes (**F**).

We hypothesized that the different TIMELESS levels found in the colon cancer cell lines could be related to the occurrence of copy number gains involving the encoding gene. To address this issue, FISH co-hybridization experiments were performed in all five colorectal cancer cell lines by using a probe set encompassing the whole gene region. The overall results showed low copy number gains of TIMELESS in all cell lines except HCT116, showing signals only on the two normal chromosome 12 homologs (Figure 6B). Specifically, HT29 displayed a whole-chromosome 12 trisomy (Figure 6D); in SW480, two markers showed positivity for all the three probes, as well as one normal chromosome 12 (Figure 6C); CaCo2, in addition to trisomy 12, showed positivity for a marker chromosome of unknown origin (Figure 6E). Finally, SW620 cells showed the most complex karyotype, displaying multiple signals to be ascribed to a whole chromosome 12 tetrasomy and two marker chromosomes positive for *TIMELESS* signals (Figure 6F).

### miR-139-5p regulates proliferation, migration, colony forming efficiency and apoptosis in human colon cancer cell lines

**Figure 7:**
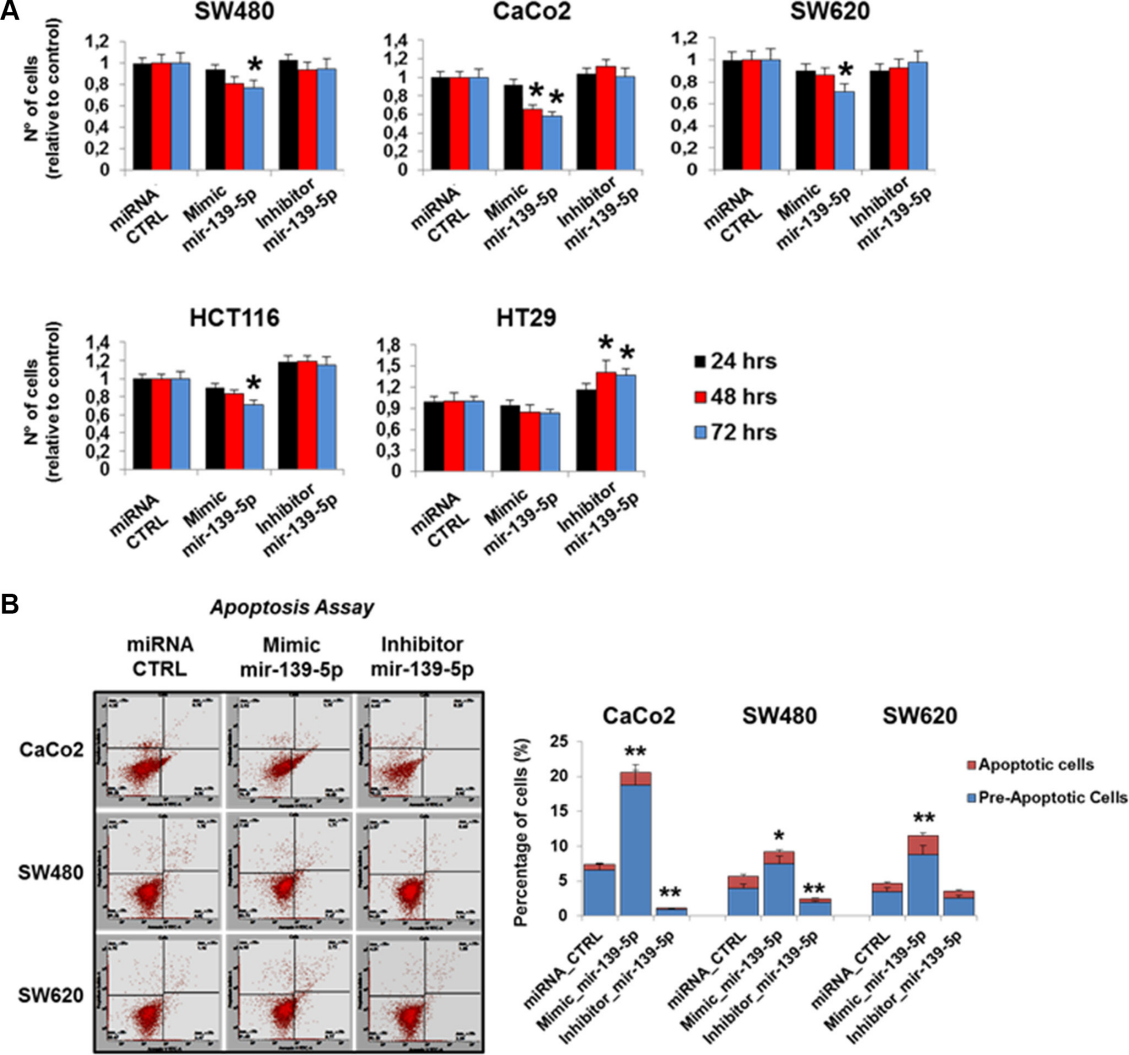
miR-139-5p inhibits cell proliferation and apoptosis in human colon cancer cells lines (**A**) Graphical representation of cell numbers assessed by automatic cell counting at different time points (24, 48 and 72 hrs) from transfection with the miR-139-5p mimic, miR-139-5p inhibitor, or the appropriate scrambled controls. (**B**) Flow cytometry analysis of pre-apoptotic and apoptotic cells (Caco-2, SW480 and SW620) transfected with the miR-139-5p mimic, inhibitor or the appropriate scrambled controls. One representative experiment out of three is shown. Samples were analyzed in triplicate and data are reported as mean ± S.D. and representative of three independent experiments; *p ≤ 0.05; **p ≤ 0.01.

**Figure 8:**
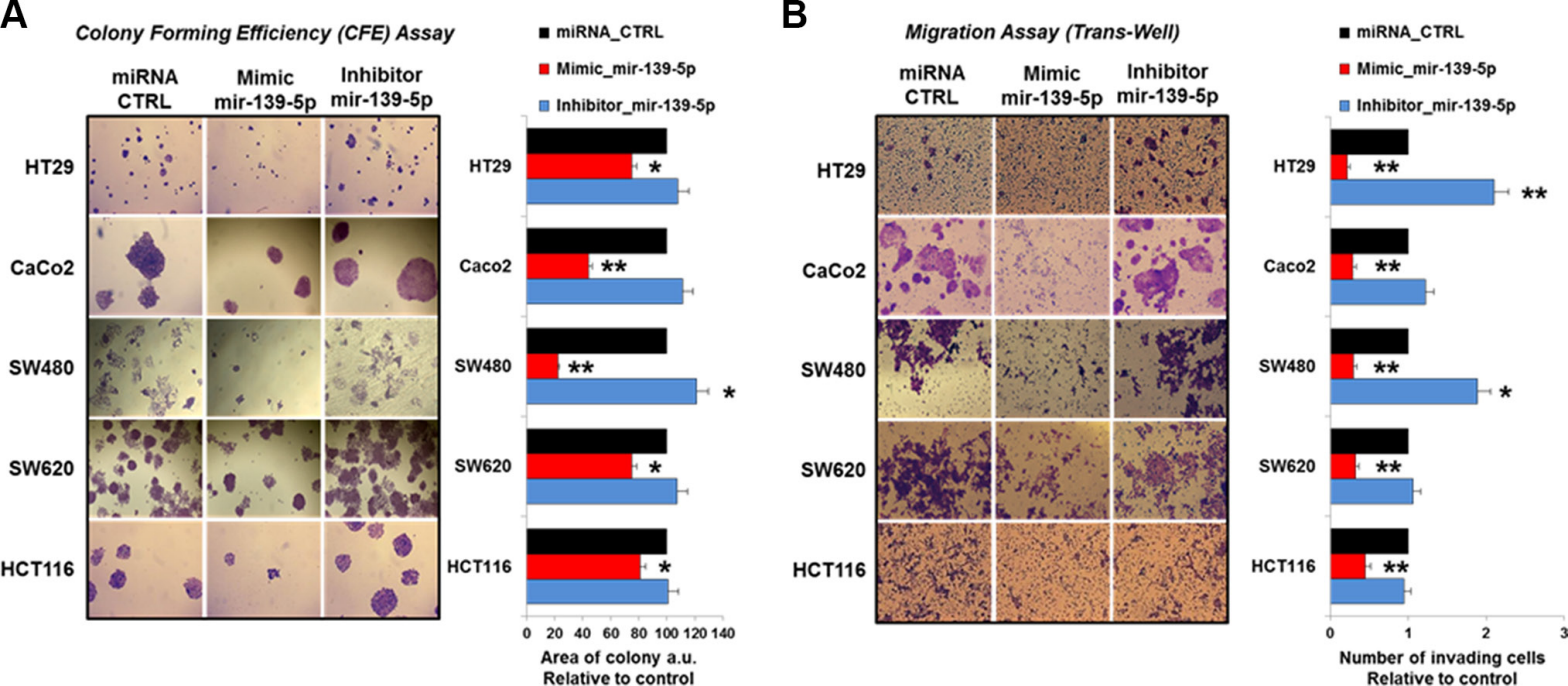
miR-139-5p reduces the colony forming area and cell invasion in human colon cancer cells lines (**A**) Plate colony-forming assay performed in the indicated cell lines transfected with miR-139-5p mimic or inhibitor or relative miRNA controls and stained with crystal violet (left panel); the histogram reports the value of colonies areas (right panel). (**B**) Number of invading cells measured in at least five fields of the trans-well migration assay in all five human colon cancer cell lines transfected as in (A). Data are reported as mean ± SD of at least four independent experiments; *p ≤ 0.05; **p ≤ 0.01.

A potential impact of TIMELESS expression changes on cancer cell phenotype modifications was suggested by data coming from the analysis of CRC patient specimens. To investigate this possibility we assessed the effects of TIMELESS down-regulation upon miR-139-5p ectopic expression on colon cancer cell proliferation, apoptosis, migration and colony forming efficiency. Cell proliferation was evaluated by automatic cell counting at different time points (24, 48 and 72 hrs) from transfection. A time-dependent reduction of proliferation was observed in all cell lines upon miR-139-5p mimic transfection (*p* < 0.05); the reduction trend was less evident in HT29 cells (Figure 7A). In contrast, due to the low basal miR-139-5p levels in all cell lines, a miR-139-5p antisense increased the proliferation rate only in HT29 cells (*p* < 0.05). Consistently, miR-139-5p overexpression or inhibition induced higher or lower pre-apoptosis and apoptosis than basal cells, respectively (Figure 7B). Colony forming efficiency and migration assays were severely reduced in all CRC cell lines examined (*p* < 0.05 and *p* < 0.01) upon miR139-5p mimic ectopic expression. In contrast, the miR-139-5p antisense induced higher migration in HT29 and SW480 cells and, in these latter ones, also larger colonies (Figure 8A and 8B).

## DISCUSSION

The definition of higher-order mRNA-miRNA interaction networks allows tumor microarray data interpretation and hypothesis generation. This, in turn, may help in discovering biological pathways deregulated in tumorigenesis and their possible roles in cancer onset and progression [28]. This method of analysis takes advantage of network-based models and, in the present study, was conducted pinpointing core clock genes and their targeting miRNAs. The data from our series of tumor and matched non-tumor tissues of CRC patients were validated with those obtained from high-throughput publicly available molecular profiling of matched colorectal tumor and non-tumor specimens.

MiRNAs play an important role in gene regulatory mechanisms negatively modulating gene expression at the post-transcriptional level. Approximately 30% of all mRNAs in mammals are regulated by miRNAs [29] and recognition occurs via base-pairing interactions with seed motifs located in the CDS or the 3′-UTR of target mRNAs. Generally, a given miRNA is expected to fine-tune a large set of mRNAs within a cell [30]. Consistently, miRNAs have been implicated in the control of genes driving proliferation, development, and differentiation [31]. Besides, miRNA expression is regulated in a circadian pattern in living beings starting from *D. melanogaster* [32] and miRNAs, in turn, modulate the biological clock [33, 34].

Interestingly, our analysis in CRC tissues shows a profound subversion of the appropriate relationships among miRNAs with respect to those detected in non-tumor tissue, with loss of connections with the main nodes and acquisition of abnormal ones with new partners that acquire key roles in the network. Among miRNAs regulating clock genes, miR-34a-5p is particularly important as it targets *CRY1* and has recently been identified as a valuable prognostic biomarker in CRC. It is, in fact, down-regulated in tumor tissues and positively correlated with disease-free survival; it is able to hinder CRC recurrence, restraining cell growth, migration and invasion, triggering cell apoptosis and cell cycle arrest in a p53-dependent manner [35]. In the non-tumor tissue network, miR-34a-5p shows the highest degree centrality as well as the lowest average shortest path length with a relatively low clustering coefficient, while in the tumor network it looses the hub function that is acquired by let-7e-5p.

This latter miRNA is implicated in colorectal carcinogenesis [36] and targets *PER1* and *CLOCK* mRNAs and in our analysis it gains a link to miR-19b-3p, which targets *TIMELESS* and *NR1D2* mRNAs. It is included in the paralogous miRNA gene cluster originating the miR-17 family miRNAs (miR-17∼92, miR-106a∼363 and miR-106b ∼ 25) involved in the control of cell cycle, proliferation, apoptosis, development and referred to as “oncomir” as frequently deregulated in hematopoietic and solid cancers [37, 38]. Finally let-7e-5p lost the link with miR-139-5p, an important tumor suppressor found deregulated in different cancer types [39–43].

miR-19b-3p is correlated with miR-99b-5p, targeting *PER1, RORA* and *TIMELESS* mRNAs and, indirectly, with miR-140-3p, targeting *NPAS2* and *TIMELESS* mRNAs. Furthermore, bioinformatic analysis identified miR-139-5p seed regions in the CDS and 3′UTR of *TIMELESS* mRNA, which plays a key role in DNA damage response, telomere length and integrity maintenance, cell cycle progression as well as processes coordinated at the DNA replication fork such as DNA synthesis, S-phase-dependent checkpoint activation and chromosome cohesion [44–47].

The potential relationship between TIMELESS and miR-139-5p was validated in our cohort of sporadic CRC patients, confirming the inverse expression pattern and suggesting that deregulation of this axis may play a role in disease progression. This was further corroborated in a series of human colon cancer cell lines in which miR-139-5p induced TIMELESS down-modulation, determining the cell phenotype. In fact, restoration of miR-139-5p significantly inhibited cell proliferation and apoptosis as evidenced by cell count and FACS analysis. In contrast, re-expression of miR-139-5p in *in vitro* assays inhibited cell migration in all five cell lines examined.

In conclusion, miRNA-miRNA interactions add complexity to the functionality and enhance the robustness of coding/noncoding RNA regulatory networks. Alteration of this multifaceted interaction plays a crucial role in carcinogenesis. Our integrative analysis of the miRNA-mRNA interplay as well as of the expression patterns of potential miRNA-miRNA pairs based on the expression profiles in CRC tissues and matched adjacent non-tumor mucosa underscores the pivotal role that the miRNA-clock genes axis plays in colorectal tumorigenesis and improves our understanding of the miRNA-mRNA regulatory networks deregulated in this common malignancy.

## MATERIALS AND METHODS

### Microarray data (subset 1)

The transcriptome and miRNAome of 14 pairs of matched tumor tissues and adjacent non-tumor mucosa obtained at surgery from CRC patients was analyzed by using the GeneChip Human Exon 1.0 ST array and the GeneChip miRNA 2.0 array (Affymetrix, Santa Clara, CA, USA), respectively [48, 49]. The clinical-pathological characteristics of the patients investigated in the current study are listed in Table 1.

### COAD-TCGA data (subset 2)

Expression profiles of COAD and healthy tissues were downloaded from the TCGA data portal, as preprocessed and normalized probeset intensity values (level 3 data).

### Selection of miRNAs targeting clock genes and miRNA symbols disambiguation

We picked up specific experimentally validated miRNAs targeting the core clock genes by the miRWalk 2.0 web service [50]. Several miRNA symbols in both the microarray and TCGA data resulted to be not updated and thus, for these, it was not possible to distinguish between the 5p from the 3p forms. We have then disambiguated and updated these symbols by a custom Python script operating on the latest CDF file of the miRNA 1.0 chip and on the list of miRNA aliases available through the miRBase web site. The only two miRNAs, i.e. miR-378 and miR-34b, which were not possible to disambiguate were kept with their original names.

### Subset and miRNA-Gene target analyses

The expression values of two miRNAs were considered coherently correlated in the tumor and normal networks if their *r* values were significant and both were either positive or negative. Any miRNA pair was tagged as *clock-controller* if at least one miRNA of the pair controlled one or more clock genes. MiRNAs were further highlighted according to their differential expression, when compared to control tissues.

### Network analysis

Networks were drawn and analyzed by Cytoscape ver. 3.2.1 (http://www.cytoscape.org/). Nodes represent miRNAs and edges represent significant correlations between two miRNAs. Attributes were added to the nodes and edges according to the following features. Nodes were colored according to their fold-changes: red for up-regulated miRNAs, green for down-regulated miRNAs and white for miRNAs with no significant or missing fold-change values. Moreover, shapes of nodes were thought to represent the co-occurrence of a miRNA in our microarray data (rounded nodes) or in the COAD-TCGA data (triangular nodes). Moreover, miRNAs targeting clock genes were marked with bold labels. Edges were colored according to their correlation levels: red for positive correlations and green for negative correlations. For each node, we calculated the following topological indices: Degree, Closeness, Centrality, Betweeness, Clustering Coefficient, Radiality and Average Shortest Path Length, as already explained elsewhere [51, 52]. In Figure 7 bigger sized nodes represented clock genes, while correlated miRNAs were linked with dashed edges.

### Validation cohort of CRC patients

The expression levels of *TIMELESS* mRNA and miR-139-5p were evaluated in the tumor tissues and adjacent non-tumor mucosa of an independent cohort of 50 CRC patients (34 men and 16 women, mean age ± SD 67.2 ± 11.6 years), surgically treated for colon and rectum primary tumors at our Hospital. The study was approved by the Ethical Board of our Institute, and all patients gave written informed consent. Patient characteristics are shown in Table 3. All tumor and non-tumor tissue specimens were collected between 9:00 a.m. and 17:00 p.m. of the same day (8 h), dissected immediately and snap-frozen in liquid nitrogen. All tissue samples were derived from surgical specimens. The frozen sections were evaluated by the pathologist to confirm the diagnosis of both tumor and non-tumor tissue. The non-tumor mucosa was collected in a part of the bowel close to the resection margins. The first and the last section of each sample were stained with haematoxylin and eosin and were analyzed by the pathologist to evaluate the tumor cell percentage. Tissue samples having at least 80% of tumor cell content were frozen in liquid nitrogen until the molecular analysis. These data were corroborated by the examination of *TIMELESS* and miR-139-5p expressions values of COAD and healthy tissues downloaded from the TCGA data portal, as preprocessed and normalized probeset intensity values (level 3).

### RNA extraction from freshly frozen tissue and colon cancer cell lines and first-strand cDNA synthesis

Preparation of total RNA from about 150–200 mg of freshly frozen tissue specimens was done using the TRIzol reagent (Invitrogen Corporation, Carlsbad, CA, USA). The amount of total extracted RNA was determined by using the Nano Drop Spectrophotometer (Nanodrop Technology, Wilmington, DE, USA), and RNA integrity was assessed using Agilent 2100 Bioanalyzer (Agilent Technologies, Santa Clara, CA, USA) after subsequent digestion by DNaseI. Next, 1.0 μg of total RNA was reversed transcribed using the High-Capacity cDNA Archive Kit following the manufacturer's instructions (Applied Biosystems, Applera, Foster City, CA, USA).

### Quantitative Real-Time reverse transcription-PCR assay

Quantitative real-time PCR (qRT-PCR) assay was used to assess the differential expression of the *TIMELESS* gene and miR-139-5p in CRC specimens matched to normal mucosa. Human QuantiTect Primers Assay (SYBR Green QuantiTect Primers Assay; QIAGEN, Hamburg, Germany) was used for *TIMELESS* (QT00019789) and qRT-PCRs were performed in a 25-μl final volume, in three replicates per sample, by using QuantiFast SYBR Green PCR kit (QIAGEN, Hamburg, Germany) and run in an ABI PRISM^®^ 7700 Sequence Detection System (Applied Biosystems, Applera, Foster City, CA, USA) according to the following conditions: 50°C for 2 min, 95°C for 10 min, and 40 cycles at 95°C for 15 s and at 60°C for 1 min. Data were acquired as threshold cycle (CT) value. Expression levels of target gene were normalized using the *GAPDH* housekeeping control gene and the relative amount of mRNA in each target gene to *GAPDH* was calculated as the average 2^−ΔΔCT^ method. Since the amplification efficiencies of target genes and internal control were equal, the relative change of target gene expression in tumor cells compared with normal colon mucosa (Δ*C*_t_ calibrator value) was calculated using the equation 2^−ΔΔ*C*t^, where ΔΔ*C*_t_ = Δ*C*_t(calibrator)_^−^Δ*C*_t(target)_. The Δ*C*_t_ values were defined by subtracting the average *GAPDH* C_t_ value from the average target gene *C*_t_ value. The SD of the difference was calculated from the SDs of target gene and *GAPDH* values. After each qRT-PCR, a melting profile as well as agarose gel electrophoresis of each sample was done to rule out nonspecific PCR products and primer dimers. For miRNA detection, 10 ng of RNA were reverse transcribed using the Taq-Man miRNA reverse transcription kit, including miR-139-5p (Assay ID: 478312_mir) and endogenous control RNU6B (Assay ID: 001093) primers in the reaction. Reverse transcription was followed by qRT-PCR assay with a TaqMan PCR Universal master mix and the appropriate miRNA specific TaqMan probe (Life Technologies Corporation). Expression levels of miR-139-5p were normalized to RNU6B and relative expression levels were calculated within each independent experiment using the formula 2^−ΔΔCT^.

### Microsatellite instability

The Microsatellite instability (MSI) analysis was performed using the Bethesda panel of microsatellite (BAT25, BAT26, D5S346, D17S250 and D2S123) evaluated by means of a multiplex-PCR and PAGE analysis. Tumors showing instability in four or more markers were classified as high MSI (MSI-H), those showing instability in two markers as low MSI (MSI-L), and those showing no instability as microsatellite-stable (MSS).

### Cell culture

CaCo2, HCT116, HT29, SW480 and SW620 cells were acquired from ATCC (American Type Cell Culture) and cultured as appropriate at 37°C in 5% CO2 atmosphere in Dulbecco's modified Eagle's medium (DMEM) and Minimum Essential Medium (MEM) Alpha media, supplemented with 10% fetal bovine serum (FBS), 100 U/ml penicillin and 100 ng/ml streptomycin (Invitrogen Life Technologies, Milan Italy).

### Immunoblot detection

CaCo2, HCT116, HT 29, SW480 and SW620 cells were lysed in a 2× Laemmli Sample buffer [250 mM Tris-HCl, pH 6.8; 500 mM DTT; 10% sodium dodecyl sulfate (SDS), 0.5% bromophenol blue and 50% glycerol) supplemented with protease inhibitor cocktail (1 mM phenylmethanesulphonylfluoride and 1 mM sodium orthovanadate). After boiling at 100°C for 3 min, equal amount of proteins were loaded on 10% polyacrylamide gels and separated by electrophoresis. Protein transfer was performed on PVDF membrane (Millipore S.p.A. Milan Italy). Membranes were blocked with 5% skim milk in wash buffer (20 mM Tris-HCl, pH 7.6, 140 mM NaCl, 0.1% Tween 20) and incubated with the specific primary antibodies diluted in blocking solution, at appropriate dilutions. Following three washes, membranes were incubated with a secondary goat anti-mouse or goat anti-rabbit horseradish peroxydase-conjugated antibody (BioRad, Hercules, CA, USA) diluted 1:3.000 in wash buffer. After three further washes, proteins were revealed by chemiluminescence (ECL, Amersham Biosciences AB, Uppsala, Sweden) and the signal detected on an X-ray film (Amersham Biosciences AB, Uppsala, Sweden). Rabbit polyclonal anti-TIMELESS antibody (ab72458) was purchased from ABCAM (Cambridge, UK). Mouse anti-β-Actin antibody (F-3022) was purchased from Sigma-Aldrich (Milan, Italy).

### Fluorescent *in situ* hybridization analysis

Each colorectal cancer cell line was tested by fluorescent *in situ* hybridization (FISH) to detect copy number gains of the chromosomal region (12q13.3) harboring the TIMELESS gene (chr12:56,810,157-56,843,200). BAC (Bacterial Artificial Chromosomes) clones encompassing both the ∼200 kb upstream and downstream regions to TIMELESS were selected according to the latest release (February 2009, GRCh37/hg19) of the University of California Santa Cruz (UCSC) Human Genome Browser (http://genome.ucsc.edu). The probes used were RP11-888B6 (chr12:56,517,568-56,727,528), RP11-348M3 (chr12:56,684,981-56,848,839) and RP11-243C3 (chr12: 56,872,287-57,047,110) (Figure 6A). Metaphase spreads were prepared as previously described [53] and FISH experiments were carried out as reported elsewhere [54]. Briefly, 600 ng of each probe were directly labeled with Cy3-, Cy5-, or FITC-conjugated dUTP by nick translation. Hybridization was performed at 37°C in 2X saline-sodium citrate (SSC), 50% (v/v) formamide, 10% (wt/vol) dextran sulfate, 5 μg Cot-1 DNA (Bethesda Research Laboratories, Gaithersburg, MD, USA), and 3 μg sonicated salmon sperm DNA in a volume of 10 μl. Three post-hybridization washes were performed at 60°C in 0.1XSSC. Chromosomes were identified by 4′,6-diamidino-2-phenylindole (DAPI) staining. Digital images were obtained using a Leica DMRXA epifluorescence microscope equipped with a cooled CCD camera (Princeton Instruments, Boston, MA, USA). Cy3 (red; New England Nuclear, Boston, MA, USA), FITC (green; Fermentas Life Sciences, Milan, Italy), Cy5 (IR; New England Nuclear), and DAPI (blue) fluorescence signals, were detected using specific filters, and recorded separately as gray-scale images. Pseudocoloring and images merging were performed by use of Adobe Photoshop software (Adobe Systems, Seattle, WA).

### Computational prediction of miR-139-5p seed regions on TIMELESS mRNA

Putative miR-139-5p binding sites in *TIMELESS* mRNA were predicted by the RNAHybrid (http://bibiserv.techfak.uni-bielefeld.de/rnahybrid/) [55] and RNA22 algorithms (https://cm.jefferson.edu/rna22/) [56] using the mature miRNA sequence reported in miRbase v.21 and the TIMELESS mature mRNA sequence found on RefSeq ad using default settings in both cases.

### Cell transfection assays

CaCo2, HCT116, HT29, SW480 and SW620 cells at 70–80% confluence were transiently transfected with synthetic miR-139–5p mimic (MSY0000250, QIAGEN) or miR-139-5p inhibitor (MIN0000250, QIAGEN) or the appropriate scrambled controls (AllStar or mirScript Inhibitor-Negative Control) using HiPerfect Transfection Reagent (QIAGEN; Hilden, Germany), following the manufacturer's instructions. Transfection efficiency was estimated by qRT-PCR evaluating miR-139-5p vs RNU6B expression levels.

### Cell proliferation assay

Cell number and density of viable cells were determined using CAsy Cell Counter (Innovatis, Sittingbourne, UK) as previously reported [57]. Briefly, each sample (cell suspension) was prepared three times in CAsyTon (Innovatis) buffer, followed by triplicate measurements of 200 μl sample volume. Viable cells were measured by Casy Cell Counter by excluding all counts that were of a size smaller that 10 μm (dead cells and debris). Three biological replicates were prepared and each assayed in triplicate and the results expressed as mean ± standard deviation (SD).

### Apoptosis assay

Apoptosis assay was performed as previously reported [58] CaCo2, SW480 and SW620 cells were transfected with miR-139-5p mimic or inhibitor or Controls 48 h at 37°C. Apoptosis was evaluated by AnnexinV-FITC and Propidium Iodide stained cells using Apoptosis Detection Kit (BD Biosciences), according to the manufacturer's protocols. All flow cytometry results were analyzed with FACSuite Software v.1.0.5.3841 (BD Biosciences). Three biological replicates were prepared and each assayed in triplicate and the results expressed as mean ± standard deviation (SD).

### Cell migration and colony forming assay

Cell migration and colony forming assay were performed as previously reported [59]. Briefly, to perform the plate colony–forming assay, 24 hours after transfection, cells were trypsinized and seeded in six-well plates (5 × 10^3^ cells); after 7 days, cells were fixed, stained and photographed under microscope. The area of single colonies was then measured by ImageJ software (National Institutes of Health Image, http://rsbweb.nih.gov/nih-image). We also performed Transwell cell migration assay. Briefly, cells after 24 h transfection were trypsinized and adjusted to 8 × 10^5^ cells/ml of cell suspension. Cells in a 200-μl volume were seeded into the upper chamber of Transwell, and added 800 μl medium with 20% FBS in the lower chamber. Cells were then cultured at 37°C for 48 h and the cells on the surface of the up chamber were swapped with cotton swap and the cells under the surface of the low chamber were stained with crystal violet (0.1%). Cells were then photographed under an inverted microscope for capturing pictures and counted for migrated cell numbers. Three biological replicates were prepared and the results expressed as mean ± standard deviation (SD).

### Statistical analysis

Raw signals of probes were background corrected, log-transformed to achieve normal distribution and then normalized using the RMA algorithm. Differential expression between samples was assessed by paired *t*-test. A miRNA was considered differentially expressed if its fold-change of expression was significantly greater than 1.5 or lower than −1.5. Correlation of expression was measured by Pearson Product Moment Correlation (PPMC) and the Pearson correlation coefficient *r* for all the probesets of tumor and non-tumor tissue. Only the pairs of human probesets with *r* significantly greater than 0.8 or lower than −0.8, were taken into account for the subsequent analysis steps. Associations between gene expression level and phenotypic characteristics were evaluated by the Pearson's chi-squared test. Survival rates were calculated by the Kaplan–Meier method for analysis of censored data. Subgroup analyses were performed after splitting the subjects into high/low expressing, based on the median value of the gene of interest. A *p*-value < 0.05 was considered statistically significant. All analyses were performed using the SPSS v17 Statistical Package (SPSS, Chicago, IL, USA), the *R* framework and Partek Genomics Suite 6.6 (Partek Inc., St. Louis, MO, USA).

## SUPPLEMENTARY MATERIALS FIGURE AND TABLES


