# Supplementary material for: Analysis of clock gene-miRNA correlation networks reveals candidate drivers in colorectal cancer
Source: Oncotarget. 2016 Jun 14;7(29):45444–61. doi: 10.18632/oncotarget.9989 (PMC5216733; doi:10.18632/oncotarget.9989)
Supplement: Supplementary file 1 [file oncotarget-07-45444-s001.pdf]

Analysis of clock gene-miRNA correlation networks reveals candidate drivers in colorectal cancer

Supplementary Materials

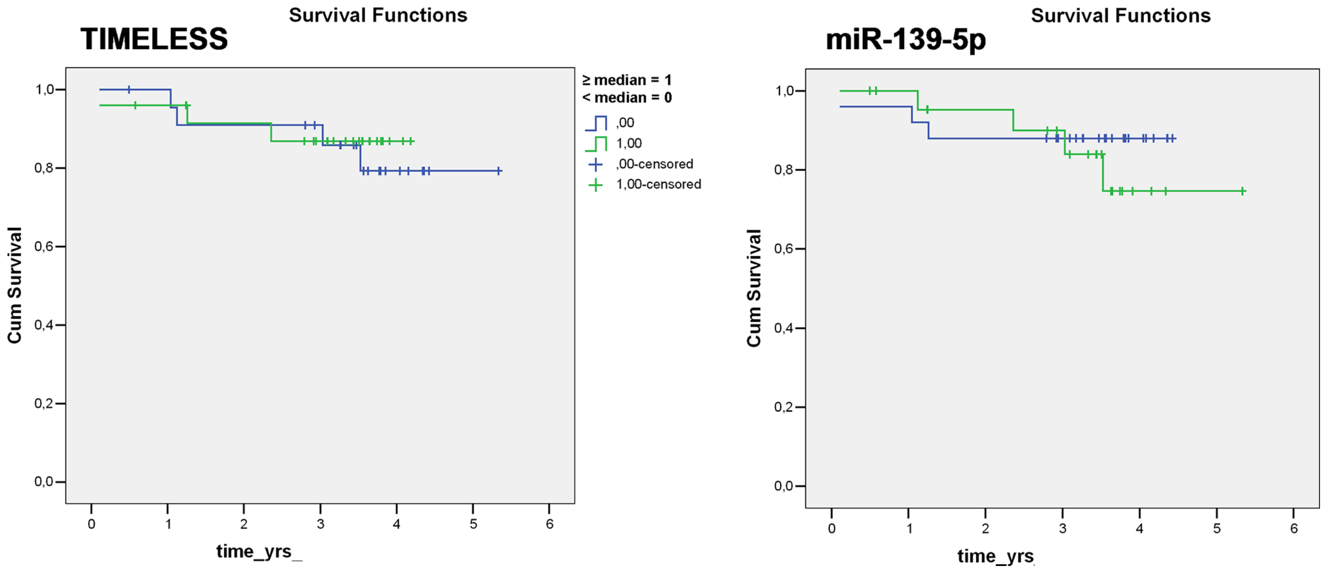

Supplementary Figure S1: Kaplan-Meier survival analysis of censored data after stratifying colorectal cancer patients on the basis of the median expression value of *TIMELESS* mRNA (left panel; Log Rank–Mantel-Cox test $p = 0.721$) and miR-139-5p (right panel; Log Rank-Mantel-Cox test $p = 0.515$).

Supplementary Table S1: Differentially expressed core clock genes in tumor tissue compared to non-tumor mucosa of colorectal cancer patients examined for the microarray analysis

Gene	Fold Change*	*P* value
ARNTL2	4.6602	1.99E-05
RORA	−2.02049	0.001935
PER1	−1.78146	0.003417
CRY2	−1.41516	0.000529
CRY1	1.58782	0.008597
NPAS2	1.79061	0.002531
TIPIN	1.79749	0.001562
CSNK1E	1.55407	0.000398
NR1D2	1.30642	0.031937
CLOCK	1.78274	0.010486
SIRT1	−1.12452	0.037362
TIMELESS	3.27267	0.004705

*Tumor versus Control.

Supplementary Table S2: Differentially expressed miRNAs in tumor tissue compared to nontumorous mucosa of colorectal cancer patients examined for the microarray analysis

	p value	FDR	Fold change
miR-378_st	0.000001	0.000489	−9.032896
miR-1246_st	0.000008	0.002352	7.076825
miR-140-3p_st	0.000006	0.002352	−7.279661
miR-139-5p_st	0.000013	0.002668	−6.801375
miR-10b_st	0.000038	0.00447	−6.100956
miR-486-5p_st	0.000044	0.00447	−6.003404
miR-378-star_st	0.000028	0.00447	−6.289438
miR-28-3p_st	0.000053	0.00447	−5.895614
miR-1826_st	0.000053	0.00447	5.895179
miR-19b_st	0.000052	0.00447	5.909628
miR-422a_st	0.000081	0.006259	−5.635207
miR-195_st	0.000108	0.007286	−5.465584
miR-342-3p_st	0.000117	0.007286	−5.418188
miR-106a_st	0.00012	0.007286	5.403466
miR-150_st	0.000149	0.008058	−5.280024
miR-17_st	0.000152	0.008058	5.267355
miR-26b_st	0.00021	0.010473	−5.08221
miR-99a_st	0.000422	0.017872	−4.691022
miR-129-3p_st	0.000413	0.017872	4.70362
miR-768-3p_st	0.000407	0.017872	−4.71143
miR-185_st	0.000463	0.018267	−4.639388
miR-27a_st	0.000474	0.018267	4.626309
miR-361-5p_st	0.000626	0.023059	−4.474231
miR-497_st	0.000718	0.023852	−4.399734
miR-21_st	0.000732	0.023852	4.389129
hsa-miR-30c-2-star_st	0.000705	0.023852	−4.409824
hsa-miR-29a_st	0.000795	0.024935	4.344628
hsa-miR-145_st	0.000865	0.026167	−4.298914
hsa-let-7e_st	0.001087	0.031761	−4.175845
hsa-miR-100_st	0.001183	0.032323	−4.130765
hsa-miR-92a_st	0.001168	0.032323	4.137504
hsa-miR-224_st	0.001222	0.032345	4.113427
hsa-miR-429_st	0.001317	0.033523	4.073522
hsa-miR-149_st	0.001346	0.033523	−4.062007
hsa-miR-92b-star_st	0.001431	0.033668	4.0293
hsa-miR-182_st	0.001402	0.033668	4.040099
hsa-miR-663_st	0.001517	0.034165	3.99832
hsa-miR-18a_st	0.001533	0.034165	3.99278
hsa-miR-30c_st	0.001754	0.037833	−3.92134
hsa-miR-363-star_st	0.001787	0.037833	−3.911554
hsa-miR-21-star_st	0.002031	0.041965	3.843773
hsa-miR-1290_st	0.002133	0.043015	3.818044
hsa-miR-143_st	0.002443	0.048114	−3.746736

FDR = false discovery rate.

Supplementary Table S3: Total number of miRNAs belonging to significantly correlated pairs

Sample	number of individual miRNAs
TCGA – Control	501
TCGA – Tumor	853
ARRAY – Control	847
ARRAY – Tumor	847

Supplementary Table S4: Presence of single miRNAs among different correlation datasets

Comparisons	Individual miRNAs present in both subsets	Individual miRNAs present only in subset 1	Individual miRNAs present only in subset 2
ARRAY-Control Vs ARRAY-Tumor	847	0	0
TCGA-Control Vs TCGA-Tumor	501	0	352
TCGA-Control vs ARRAY-Control	253	248	594
TCGA-Tumor vs ARRAY-Tumor	401	452	446

Supplementary Table S5: Amount of miRNAs targeting core clock genes among the different miRNA subsets

Pairs in subsets			
**Array**	**Only 1 member in Pair**	**Both Members in Pair**	**Total**
Common Pairs between Tumor and Control	191	63	254
Pairs Belonging to Control Only	1635	173	1808
Pairs Belonging to Tumor Only	1172	199	1371

TCGA	Only 1 member in Pair	Both Members in Pair	Total
Common Pairs between Tumor and Control	28	3	31
Pairs Belonging to Control Only	1422	62	1484
Pairs Belonging to Tumor Only	146	15	161

Supplementary Table S6: IPA functions for gene/miRna subsets

Functions	Diseases or Functions Annotation	*p*-value	Predicted Activation State	Activation z-score	Molecules
proliferation	proliferation of cells	3.60E-04	Increased	2.191	let-7e-5p (and other miRNAs w/seed GAGGUAG),miR-100-5p (and other miRNAs w/seed ACCCGUA),miR-125b-5p (and other miRNAs w/seed CCCUGAG), miR-139-5p (miRNAs w/seed CUACAGU),miR-17-5p (and other miRNAs w/seed AAAGUGC),miR-18a-5p (and other miRNAs w/seed AAGGUGC), miR-19b-3p (and other miRNAs w/seed GUGCAAA), miR-92a-3p (and other miRNAs w/seed AUUGCAC),RORA,TIMELESS
proliferation	proliferation of carcinoma cell lines	1.05E-04	Increased	1.987	let-7e-5p (and other miRNAs w/seed GAGGUAG),miR-17-5p (and other miRNAs w/seed AAAGUGC),miR-18a-5p (and other miRNAs w/seed AAGGUGC),miR-19b-3p (and other miRNAs w/seed GUGCAAA)
growth	growth of tumor	4.31E-03	Increased	1.969	miR-100-5p (and other miRNAs w/seed ACCCGUA),miR-125b-5p (and other miRNAs w/seed CCCUGAG),miR-17-5p (and other miRNAs w/seed AAAGUGC),RORA
proliferation	proliferation of tumor cell lines	1.80E-05	Increased	1.789	let-7e-5p (and other miRNAs w/seed GAGGUAG),miR-100-5p (and other miRNAs w/seed ACCCGUA),miR-125b-5p (and other miRNAs w/seed CCCUGAG),miR-139-5p (miRNAs w/seed CUACAGU),miR-17-5p (and other miRNAs w/seed AAAGUGC),miR-18a-5p (and other miRNAs w/seed AAGGUGC),miR-19b-3p (and other miRNAs w/seed GUGCAAA),RORA
invasion	invasion of cells	5.68E-03	Increased	1.228	miR-125b-5p (and other miRNAs w/seed CCCUGAG),miR-17-5p (and other miRNAs w/seed AAAGUGC),miR-196a-5p (and other miRNAs w/seed AGGUAGU),RORA
